# Antifeedant and Antiviral Diterpenoids from the Fresh Roots of *Euphorbia jolkinii*

**DOI:** 10.1007/s13659-014-0009-3

**Published:** 2014-04-09

**Authors:** Chun-Shuai Huang, Shi-Hong Luo, Yao-Lan Li, Chun-Huan Li, Juan Hua, Yan Liu, Shu-Xi Jing, Ying Wang, Min-Jie Yang, Sheng-Hong Li

**Affiliations:** 1 State Key Laboratory of Phytochemistry and Plant Resources in West China, Kunming Institute of Botany, Chinese Academy of Sciences, Kunming, 650201 China; 2 Institute of Traditional Chinese Medicine and Natural Products, Jinan University, Guangzhou, 510632 China; 3 Graduate University of Chinese Academy of Sciences, Beijing, 100049 China

**Keywords:** *Euphorbia jolkinii*, Diterpenoids, Antifeedant activity, Antiviral activity

## Abstract

The perennial herbaceous plant *Euphorbia jolkinii* (Euphorbiaceae) is a noxious weed widely distributed in the grasslands of northwestern Yunnan and has greatly threatened the local biodiversity. Phytochemical investigation on the fresh roots of *E. jolkinii* afforded six new diterpenoids **1**, **2**, **4**–**6**, and **8**, together with fifteen known diterpenoids. Their structures were elucidated on the basis of 1D and 2D NMR and other spectroscopic methods. Casbane, lathyrane, abietane, and *ent*-kaurane diterpenoids were reported from this plant for the first time. Selected compounds were evaluated for their antifeedant and anti-RSV (respiratory syncytial virus) activities. Compound **2** and ingenol (**3**) exhibited moderate antifeedant activity against a generalist insect herbivore, *Spodoptera exigua*, with EC_50_ values of 17.88 and 17.71 μg/cm^2^ respectively. Compound **19** showed significant anti-RSV activity, with 50 % inhibition (IC_50_) value of 10.0 μM and selective index of 8.0. Compounds **1** and **2** were less active against RSV virus, both with IC_50_ value of 25 μM, and with selective indices of 1.0 and 3.2 respectively. These findings provided new evidence for the biological functions and utilization of the diversified diterpenoid metabolites in the roots of this rich but harmful plant.

**Electronic supplementary material:**

The online version of this article (doi:10.1007/s13659-014-0009-3) contains supplementary material, which is available to authorized users.

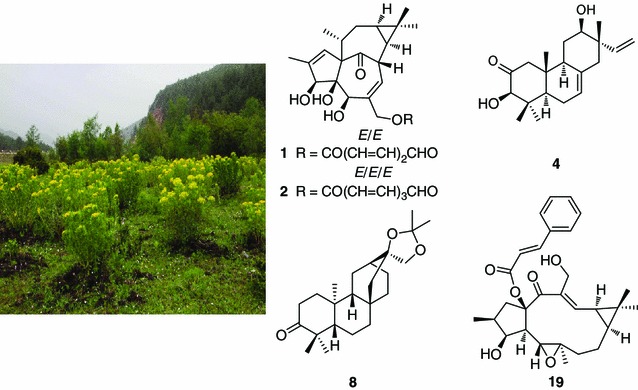

## Introduction

*Euphorbia* is the largest genus of Euphorbiaceae which contains more than 2000 species, mainly distributing in the tropical and subtropical regions of Africa and America [1]. Extensive phytochemical investigations of different *Euphorbia* species have been conducted, therefore it has been proved to be a rich source of diterpenoids with diverse structures and interesting biological activities [2–4].

There are more than 80 *Euphorbia* species in China [5], widely distributed throughout the whole country especially in the southwestern Hengduan Mountains and northwestern arid regions. *Euphorbia jolkinii* (or *Euphorbia nematocypha*), a perennial herbaceous plant, is indigenous to Southwest of China and particularly rich in the grasslands of northwestern Yunnan. This plant has a highly developed root system that could extend 1–2 m far away from where they thrived. It is harmful to local agriculture and animal husbandry, which has caused serious grassland degradation and ecological disasters in its distribution area. Previous phytochemical investigations on *E. jolkinii* have led to the isolation and identification of a number of terpenoid secondary metabolites [6–8], most of which exhibited only weak or no biological activities. To understand the biological functions of the secondary metabolites of *E. jolkinii*, we have carried out a detailed investigation on the chemistry of the fresh roots of this plant. Herein, we described the isolation and structural elucidation of six new and 15 known diterpenoids from *E. jolkinii*, and the antifeedant and antiviral activities of some of these compounds.

## Results and Discussion

**Fig. 1:**
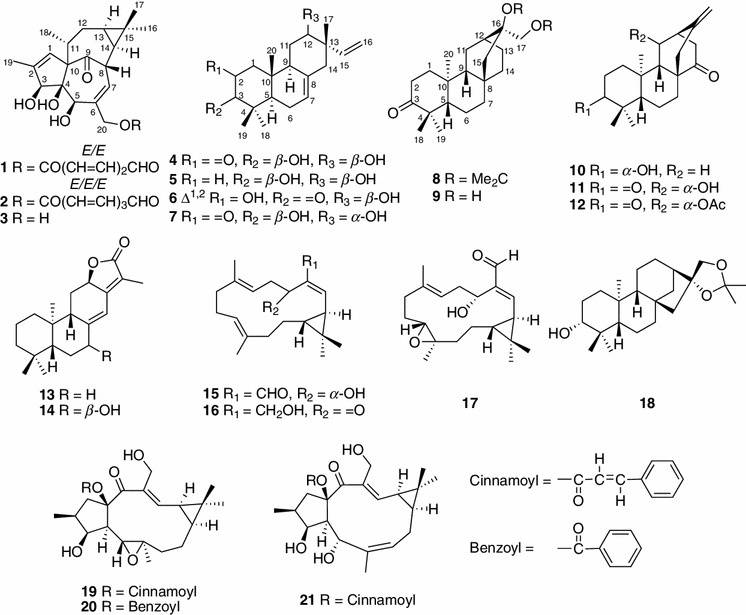
Chemical structures of compounds **1-21**

**Fig. 2:**
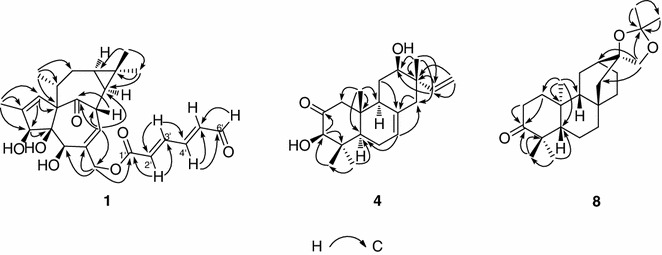
Selected HMBC (*arrow*) correlations of compounds **1**, **4**, and **8**

Compound **1** has a molecular formula C_26_H_32_O_7_, as deduced from the HR-EI-MS (found: *m/z* 456.2169; calcd. for 456.2148), requiring eleven degrees of unsaturation. The presence of hydroxyl groups (3434 cm^−1^), carbonyl groups (1719 and 1686 cm^−1^), and olefinic groups (1637 cm^−1^) were clearly shown in the IR spectrum of **1**. The UV maximum at 267 nm and MS fragmental peak at *m/z* 330 [M-CHO(CH=CH)_2_COOH]^−^ indicated the existence of a (2*E*,4*E*)-6-*oxo*-2,4-hexadienoyloxy group, which was supported by five sequentially coupled protons at *δ*_H_ 6.48 (d, *J* = 14.3 Hz), 7.46 (dd, *J* = 14.3, 11.2 Hz), 7.44 (dd, *J* = 14.4, 11.2 Hz), 6.51 (dd, *J* = 14.4, 7.8 Hz), and 9.70 (d, *J* = 7.8 Hz) in the ^1^H NMR spectrum of **1** (Table 1). In addition, the ^1^H NMR signals suggested the presence of a secondary methyl at *δ*_H_ 0.92 (d, *J* = 7.0 Hz) and three tertiary methyls at *δ*_H_ 1.04, 1.10, and 1.80, two olefinic protons at *δ*_H_ 5.81 (s) and 6.04 (d, *J* = 3.9 Hz), two oxy-methines at *δ*_H_ 3.62 (s) and 4.43 (br. s), and one oxy-methylene at *δ*_H_ 4.63 (ABd, *J* = 12.8 Hz) and 4.84 (ABd, *J* = 12.8 Hz). In the relatively high field, two protons at *δ*_H_ 0.66 and 0.85 were indicative of a cyclopropyl residue. The ^13^C NMR spectrum of **1** exhibited 26 carbons. Besides the signals for the above-mentioned (2*E*,4*E*)-6-*oxo*-2,4-hexadienoyloxy moiety, there were still 20 carbons which were classified as four methyls, two methylenes including an oxymethylene, eight methines including two oxymethines and two olefinic methines, and six quaternary carbons including an oxygenated one, two olefinic ones, and a keto. The two methines at *δ*_C_ 23.9 and 24.1 and the quaternary carbon at *δ*_C_ 24.4 further supported the existence of a cyclopropyl residue. The molecular formula of **1** still needed three degrees of unsaturation, indicating three additional rings were available in **1**. These spectral evidences indicated that compound **1** was a tetracyclic diterpenoid containing a cyclopropane in its skeleton and a six-carbon ester side chain as its substituent. Comparison of the ^1^H and ^13^C NMR spectra of **1** with those of 20-*O*-(2′*E*,4′*E*-decadienoyl) ingenol [9] and sikkimenoid E [10], which were isolated from *Euphorbia kansui* and *Euphorbia sikkimensis* respectively, indicated that **1** was very similar to these two compounds, with the only difference in their C-20 ester side chains. The decadienoyl group in 20-*O*-(2′*E*,4′*E*-decadienoyl) ingenol and sikkimenoid E was found to have been replaced by a (2*E*,4*E*)-6-*oxo*-2,4-hexadienoyl group in **1**, which was confirmed by the 2D NMR experiments (^1^H–^1^H COSY, HSQC, and HMBC) of **1** (for selected HMBC correlations, see Fig. 2). The ROESY spectrum of **1** indicated that the configurations of the chrial centers in the skeleton of **1** were identical to those of 20-*O*-(2′*E*,4′*E*-decadienoyl) ingenol and sikkimenoid E. Therefore, compound **1** was concluded to be 20-*O*-[(2′*E*,4′*E*)-6-*oxo*-2′,4′-hexadienoyl] ingenol (Fig. 1).

**Table 1:** ^1^H and ^13^C NMR data for compounds **1**, **2**, and **4** in acetone-*d*_6_ (*δ* in ppm, *J* in Hz)

No.	**1**		**2**		**4**	
	*δ*_H_	*δ*_C_	*δ*_H_	*δ*_C_	*δ*_H_	*δ*_C_
1	5.81 s	129.5 d	5.81 s	129.5 d	2.47 m	51.6 t
2		140.3 s		140.3 s		210.9 s
3	4.43 br. s	80.3 d	4.45 d (6.1)	80.3 d	4.05 d (5.2)	82.6 d
4		85.4 s		85.4 s		45.4 s
5	3.62 s	74.4 d	3.62 d (10.7)	74.4 d	1.88 m	49.4 d
6		138.5 s		138.6 s	2.07 m (2H)	24.0 t
7	6.04 d (3.9)	127.5 d	6.02 d (4.2)	127.2 d	5.46 br. s	122.4 d
8	4.20 dd (12.0, 4.0)	44.6 d	4.20 dd (11.9, 3.3)	44.6 d		135.0 s
9		206.0 s		206.0 s	2.24 m	51.4 d
10		73.4 s		73.3 s		42.4 s
11a	2.44 m	39.8 d	2.42 m	39.9 d	1.63 m (*α*)	29.5 t
11b					1.37 m (*β*)	
12a	2.36 m	30.4 t	2.35 m	31.5 t	3.52 ddd (11.6, 4.4, 4.0)	74.6 d
12b	1.70 m		1.72 m			
13	0.66 m	23.9 d	0.66 m	23.9 d		42.7 s
14a	0.85 dd (11.8, 8.5)	24.1 d	0.85 dd (11.8, 8.5)	24.1 d	2.09 m	45.4 t
14b					1.97 d (14.8)	
15		24.4 s		24.4 s	5.94 dd (17.6, 10.8)	148.3 d
16a	1.04 s (3H)	28.7 q	1.04 s (3H)	28.7 q	5.01 dd (17.6, 1.4)	112.0 t
16b					4.96 dd (10.8, 1.4)	
17	1.10 s (3H)	15.8 q	1.10 s (3H)	15.8 q	0.84 s (3H)	15.1 q
18	0.92 d (7.0, 3H)	17.5 q	0.92 d (7.0, 3H)	17.5 q	1.13 s (3H)	28.8 q
19	1.80 s (3H)	15.5 q	1.81 s (3H)	15.5 q	0.75 s (3H)	16.8 q
20a	4.84 d (12.8)	67.4 t	4.81 d (12.9)	67.1 t	0.88 s (3H)	15.8 q
20b	4.63 d (12.8)		4.63 d (12.7)			
1′		165.7 s		166.3 s		
2′	6.48 d (14.3)	130.4 d	6.21 d (15.3)	126.3 d		
3′	7.46 dd (14.3, 11.2)	141.7 d	7.35 m	143.4 d		
4′	7.44 dd (14.4, 11.2)	148.4 d	6.99 m	139.4 d		
5′	6.51 dd (14.4, 7.8)	138.0 d	6.97 m	138.6 d		
6′	9.70 d (7.8)	194.1 d	7.39 m	150.8 d		
7′			6.31 dd (15.2, 7.9)	134.9 d		
8′			9.65 d (7.9)	194.0 d		
3-OH			5.22 d (6.3)		3.70 d (5.2)	
4-OH			4.61 s			
5-OH			3.98 d (11.3)			
12-OH					3.44 d (4.4)	

^1^H and ^13^C NMR were recorded at 400 and 100 MHz, respectively

**Fig. 3:**
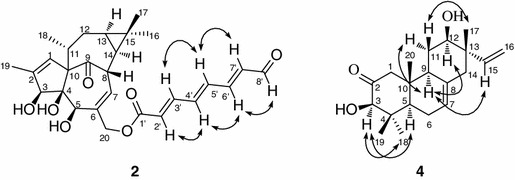
Key ROESY correlations of **2** and **4**

The molecular formula of compound **2** was established as C_28_H_34_O_7_ through an analysis of its HR-EI-MS (found: *m/z* 482.2301; calcd. for 482.2305). The ^1^H and ^13^C NMR spectral data attributable to the diterpenoid skeleton of **2** (Table 1) were almost same with those of **1**. The main difference was that two additional olefinic methines appeared at the downfield in the ^13^C NMR spectrum of **2**, suggesting that the 6-*oxo*-2,4-hexadienoyloxy group in **1** was replaced by a 8-*oxo*-2,4,6-octatrienoyloxy group in **2**. The coupling constants of those olefinic protons (*J*_2′,3′_ = 15.3 Hz; *J*_6′,7′_ = 15.2 Hz) indicated that both the configurations of Δ^2′,3′^ and Δ^6′,7′^ were *E*. Although H-4′ and H-5′ were partially overlapped and their couplings were complicated, the *E* configuration of Δ^4′,5′^ could be determined by the ROESY interactions between H-3′ and H-5′ and between H-4′ and H-6′ (Fig. 3). Ultimately, the structure of **2** was identified as 20-*O*-[(2′*E*,4′*E*,6′*E*)-8-*oxo*-2′,4′,6′-octatrienoyl] ingenol (Fig. 1).

Compound **4** was isolated as colorless needles, and its molecular formula was determined to be C_20_H_30_O_3_ on the basis of the HR-EI-MS (found: *m/z* 318.2194; calcd. for 318.2195). Its IR absorption at 3442, 1716, and 1632 cm^−1^ showed the presence of hydroxyl group, carbonyl group, and olefinic group. In the ^1^H NMR spectrum of **4**, four tertiary methyls at *δ*_H_ 0.75, 0.84, 0.88, and 1.13 were clearly shown (Table 1). Two proton signals resonated from oxygenated methines were present at *δ*_H_ 3.52 (ddd, *J* = 11.6, 4.4, 4.0 Hz) and 4.05 (d, *J* = 5.2 Hz). In addition, an *ABX* system for a vinyl group at *δ*_H_ 4.96 (dd, *J* = 10.8, 1.4 Hz), 5.01 (dd, *J* = 17.6, 1.4 Hz), and 5.94 (dd, *J* = 17.6, 10.8 Hz) and an additional olefinic proton at *δ*_H_ 5.46 (brs) were observed. In the ^13^C NMR spectrum of **4**, twenty carbon signals were exhibited, which were further classified by DEPT-90 and DEPT-135 experiments as four methyls, five methylenes including a terminal olefinic methylene (*δ*_C_ 112.0), six methines including two oxygenated methines (*δ*_C_ 74.6 and 82.6) and two olefinic methines (*δ*_C_ 122.4 and 148.3), and five quaternary carbons including an olefinic one (*δ*_C_ 135.0) and a keto (*δ*_C_ 210.9). The above spectral evidences, in combination with a comparison of the NMR spectra of **4** with those of a known isopimarane diterpenoid that was isolated from *E. jolkinii*, (3*β*,12*α*)-3,12-dihydroxyisopimara-7,15-dien-2-one [8], revealed that the two compounds were extremely similar. The ^1^H–^1^H COSY, HSQC, and HMBC (Fig. 2) experiments of **4** confirmed that its planar structure was actually identical to (3*β*,12*α*)-3,12-dihydroxyisopimara-7,15-dien-2-one. The only difference between these two compounds was that the oxygenated methine (C-12) at *δ*_C_ 72.3 in (3*β*,12*α*)-3,12-dihydroxyisopimara-7,15-dien-2-one [8] shifted downfield to *δ*_C_ 74.6 in **4**, and concurrent chemical shift changes of those carbons surrounding C-12 also occurred. In the ROESY spectra of **4** (Fig. 3), the correlations of H-3 with Me-18 and H-5 indicated that H-3 was remained to be *α*-oriented. Moreover, the correlation of Me-17 with H-11*β* elucidated a *β*-orientation for Me-17, thus confirmed the isopimarane skeleton of **4**. However, the correlations of H-12 with H-9 and H-11*α* indicated that H-12 was in *α*-orientation, which was on the contrary to that of (3*β*,12*α*)-3,12-dihydroxyisopimara-7,15-dien-2-one [8]. Finally, compound **4** was characterized as (3*β*,12*β*)-3,12-dihydroxyisopimara-7,15-dien-2-one (Fig. 1).

Compound **5** was also obtained as colorless needles. The molecular ion peak at *m/z* 304.2401 in HR-EI-MS showed a molecular formula of C_20_H_32_O_2_ (calcd. for 304.2402). The close resemblance between the NMR spectra of **5** (Table 2) and those of **4** indicated that **5** was also an isopimarane diterpenoid. The major difference between them was that the keto group in **4** disappeared while one more methylene carbon (*δ*_C_ 28.4) appeared in **5**. Based on ^1^H-^1^H COSY correlations of H_2_-1 and H-3 with the methylene protons, it was elucidated that this menthylene was ascribable to C-2. The relative configuration of all chiral centers in **5** remained unchanged, as was established by a 2D ROESY experiment. The ROEs of H-3 with Me-18 and H-5, of 3-OH with Me-19, and of H-14*β* with 12-OH, indicated that both H-3 and H-12 were in *α*-orientation. Accordingly, the structure of compound **5** was identified as (3*β*,12*β*)-3,12-dihydroxyisopimara-7,15-diene (Fig. 1).

**Table 2:** ^1^H and ^13^C NMR data for compounds **5**, **6**, and **8** (*δ* in ppm, *J* in Hz)

No.	**5** ^a,b^		**6** ^c,d^		**8** ^b,c^	
	*δ*_H_	*δ*_C_	*δ*_H_	*δ*_C_	*δ*_H_	*δ*_C_
1a	1.77 m (*β*)	38.5 t	6.21 s	123.6 d	1.85 m	38.7 t
1b	1.14 m (*α*)				1.37 m	
2a	1.56 m (2H)	28.4 t		143.6 s	2.60 ddd (15.9, 12.4, 6.8)	34.4 t
2b					2.24 ddd (15.9, 6.0, 3.2)	
3	3.19 dt (10.6, 4.8)	78.8 d		200.5 s		215.6 s
4		39.4 s		43.4 s		48.0 s
5	1.16 m	51.1 d	1.86 dd (11.6, 3.6)	48.0 d	1.34 m	56.3 d
6a	1.96 m (2H)	23.9 t	2.09 m (*β*)	22.7 t	1.53 m (2H)	20.2 t
6b			1.98 m (*α*)			
7a	5.39 dd (4.8, 2.4)	121.9 d	5.51 d (4.8)	122.6 d	1.42 m	39.8 t
7b					1.19 m	
8		136.3 s		133.4 s		33.8 s
9	2.12 m	46.5 d	2.10 m	47.6 d	1.25 m	51.4 d
10		35.7 s		36.3 s		37.9 s
11a	1.66 m (2H)	28.1 t	1.93 m (*α*)	27.2 t	1.99 m	23.7 t
11b			1.45 m (*β*)		1.27 m	
12	3.59 br. s	73.6 d	3.54 dd (12.0, 4.4)	73.7 d	1.67 m	35.2 d
13		42.0 s		42.7 s	1.54 m	23.8 t
14a	2.57 d (13.2, *β*)	39.7 t	2.03 m (2H)	45.4 t	1.89 m	27.8 t
14b	1.70 d (13.2, *α*)				0.87 m	
15a	6.01 dd (17.4, 10.8)	148.3 d	5.75 dd (17.4, 10.8)	146.0 d	1.50 m	54.9 t
15b					1.40 m	
16a	4.97 dd (17.4, 1.2)	111.8 t	5.15 dd (17.4, 1.2)	114.6 t		83.0 s
16b	4.95 dd (10.8, 1.2)		5.17 dd (10.8, 1.2)			
17a	0.85 s (3H)	22.6 q	0.91 s (3H)	13.7 q	3.95 d (8.4)	75.0 t
17b					3.62 d (8.4)	
18	0.98 s (3H)	29.0 q	1.20 s (3H)	25.2 q	1.03 s (3H)	26.4 q
19	0.89 s (3H)	16.3 q	1.16 s (3H)	22.4 q	1.01 s (3H)	21.8 q
20	0.87 s (3H)	15.2 q	1.11 s (3H)	15.8 q	1.14 s (3H)	13.8 q
1′						109.1 s
2′					1.29 s (3H)	27.4 q
3′					1.28 s (3H)	27.7 q
2-OH			6.05 br. s			
3-OH	3.51 d (4.8)					
12-OH	3.39 d (3.6)					

^a^^1^H and ^13^C NMR were recorded at 600 and 150 MHz, respectively

^b^Measured in acetone-*d*_6_

^c^^1^H and ^13^C NMR were recorded at 400 and 100 MHz, respectively

^d^Measured in CDCl_3_

Compound **6** was attributed a molecular formula of C_20_H_28_O_3_ by its HR-EI-MS (found: *m/z* 316.2038; calcd. for 316.2038), corresponding to seven degrees of unsaturation. Analysis of its NMR spectral data (Table 2) disclosed that **6** was again an isopimarane diterpenoid whose structure was similar to **4**. However, it was noteworthy that an additional tri-substituted double bond was found to have replaced a methylene and an oxygenated methine in **6**. In addition, the keto carbon in **6** was shifted dramatically upfield by 10.4 ppm, suggesting that this keto group was conjugated. In the HMBC spectrum of **6**, correlations from Me-19 (*δ*_H_ 1.16) and Me-18 (*δ*_H_ 1.20) to the keto carbon at *δ*_C_ 200.5 were observed, which allowed the location of this keto group to be at C-3, thus also suggested that the additional tri-substituted double bond should be formed between C-1 and C-2. The HMBC cross-peaks from the deshielded olefinic singlet *δ*_H_ 6.21 (H-1) to C-20 (*δ*_C_ 15.8), C-10 (*δ*_C_ 36.3), C-5 (*δ*_C_ 48.0), C-3 (*δ*_C_ 200.5), and C-2 (*δ*_C_ 143.6), and from the broad singlet at *δ*_H_ 6.05 (2-OH) to C-1 (*δ*_C_ 123.6) indicated that C-2 of **6** was hydroxylated. The ROESY spectrum of **6** showed identical correlations to those for **4**. Consequently, compound **6** was determined as (12*β*)-2,12-dihydroxy isopimara-1,7,15-trien-3-one (Fig. 1).

Compound **8** was assigned a molecular formula of C_23_H_36_O_3_, as determined by its HR-EI-MS (found: *m/z* 360.2682; calcd. for 360.2664), indicating six degrees of unsaturation. The IR spectrum showed the existence of carbonyl group at 1706 cm^−1^. The ^1^H NMR spectrum (Table 2) showed five tertiary methyls at *δ*_H_ 1.01, 1.03, 1.14, 1.28, and 1.29, and a pair of AB doublets at *δ*_H_ 3.62 and 3.95 owing to an oxymethylene. The ^13^C NMR and DEPT spectra (Table 2) indicated the presence of a keto carbon (*δ*_C_ 215.6) and 22 *sp*^3^ carbons separately for five methyls, nine methylenes including an oxy-methylene (*δ*_C_ 75.0), three methines, and six quaternary carbons including an oxygenated one (*δ*_C_ 83.0) and a ketal carbon (*δ*_C_ 109.1). These NMR data were very similar to those of *ent*-l6*α*,17-dihydroxyatisan-3-one that was isolated from *E. fidjiana* [11], implying that **8** was also an *ent*-atisane diterpenoid but containing an acetonide. This acetonide could only be formed between C-17 and C-16, as was supported by the downfield chemical shifts of C-16 (*δ*_C_ 83.0) and C-17 (*δ*_C_ 75.0) and the HMBC correlations from H_2_-17 to the ketal carbon (Fig. 2). Thus, the structure of compound **8** was assigned as *ent*-3-oxoatis-16*α*,17-acetonide (Fig. 1).

Fifteen known diterpenoids, namely, ingenol (**3**) [12], (3*β*,12*α*)-3,12-dihydroxyisopimara-7,15-dien-2-one (**7**) [8], *ent*-l6*α*,17-dihydroxyatisan-3-one (**9**) [11], *ent*-(3*α*,5*β*,8*α*,9*β*,10*α*,12*α*)-3-hydroxyatis-16-en-14-one (**10**) [8], *ent*-(5*β*,8*α*,9*β*,10*α*,11*α*,12*α*)-11-hydroxyatis-16-ene-3,14-dione (**11**) [8], *ent*-(5*β*,8*α*,9*β*,10*α*,11*α*,12*α*)-3,14-dioxoatis-16-en-11-yl acetate (**12**) [8], jolkinolide E (**13**) [13, 14], 7*β*-hydroxy-*ent*-abieta-8(14),13(15)-dien-12*α*,16-olide (**14**) [15], pekinenal (**15**) [16], pekinenin A (**16**) [17], pekinenin D (**17**) [18], *ent*-l6*β*,17-isopropylidenedioxy kauran-3*β*-ol (**18**) [19], jolkinol A (**19**) [20], jolkinol A′ (**20**) [20], and 3*β*,5*α*,20-trihydroxy-15*β*-cinnamoyloxy-14-oxolathyra-6*Z*,12*E*-diene (**21**) [21], were also isolated and identified by comparison of their spectroscopic data with those reported values in the literatures.

The antifeedant activity of compounds **2**, **3**, and **15** against the larvae of a generalist insect herbivore, the beet armyworm (*Spodoptera exigua*), were tested as previously described [22, 23]. It was found that compounds **2** and **3** showed moderate deterrence against the insect, with EC_50_ values of 17.88 and 17.71 μg/cm^2^, respectively (Table 3), which were less active than the commercial neem oil (1 % azadirachtin, EC_50_ = 3.71 μg/cm^2^) by Kunming Rixin Dachuan Technology Co., Ltd. Compounds **1**–**3**, **10**, **11**, **13**, **15**, and **19**, were assayed for their anti-RSV activity according to the reported method [24, 25]. Compound **19** showed significant anti-RSV activity, with IC_50_ value of 10.0 μM and selective index of 8.0. Compounds **1** and **2** were less active against RSV, both having IC_50_ value of 25.0 μM, and SI of 1.0 and 3.2 respectively (Table 4). However, the other compounds were basically inactive. Compounds **10**, **13**, and **15** were also evaluated for their antiviral activities against Herpes simplex type one (HSV-1) and Coxsackie B3 (Cox B3) viruses as described in the literature [24], but none of them showed obvious activity.

**Table 3:** Antifeedant activity of compounds **2**, **3**, and **15**

Compounds	Antifeedant activity^a^
**2**	17.88
**3**	17.71
**15**	79.27
Neem oil (1 % azadirachtin)	3.71

^a^Results of antifeedant activity against *Spodoptera exigua* were expressed as EC_50_ (μg/cm^2^) values

**Table 4:** Anti-RSV activity, cytotoxicity, and selective index of compounds **1**, **2**, and **19**

Compounds	Anti-RSV activity^a^ IC_50_ (μM)^b^	Cytotoxic effect CC_50_ (μM)^c^	Selective index (SI)^d^
**1**	25.0	25.0	1.0
**2**	25.0	80.0	3.2
**19**	10.0	80.0	8.0
Ribavirin	6.97	256.0	36.7

^a^The data of anti-RSV activity were measured by plaque reduction assay

^b^IC_50_: the concentration of the sample required to inhibit 50 % virus-induced Cytopathic effects (CPE)

^c^CC_50_: the concentration of the 50 % cytotoxic effect

^d^SI: the ratio CC_50_/IC_50_

In summary, we have isolated and identified six new and fifteen known diterpenoids from the fresh roots of *E. jolkinii*. These compounds belong to seven different types of skeletons, including ingenane, isopimarane, *ent*-atisane, casbane, lathyrane, abietane, and *ent*-kaurane. Among them, the diterpenoids with casbane, lathyrane, abietane, and *ent*-kaurane skeletons were reported from this plant for the first time, which further broadened our knowledge of the diversified diterpenoid metabolites in *E. jolkinii*. The antifeedant, antiviral, and cytotoxic activities of these diterpenoids implied their possible roles in the plant as constitutive defense compounds against insects, animals, and microbes, and provided new evidence for the exploitation of this rich but harmful plant, which are worthy of more future investigations.

## Experimental Section

### General Experimental Procedures

Optical rotations were determined on a Horiba-SEAP-300 spectropolarimeter. UV spectral was obtained on a Shimadzu-210A double-beam spectrophotometer. IR spectra were recorded on a Bruker-Tensor-27 spectrometer with KBr pellets. ^1^H NMR, ^13^C NMR, and 2D NMR spectra were taken on either a Bruker AM-400, DRX-500 or Bruker Avance III 600 spectrometers with TMS as internal standard. EI-MS and HR-EI-MS data were measured on a VG-Auto-Spec-3000 spectrometer. Semipreparative HPLC was carried out on an Agilent 1200 series instrument equipped with a quaternary pump, a vacuum degasser, an autosampler, a thermostatic column compartment, and a diode array detector. Column chromatography was performed with silica gel (200–300 mesh, Qingdao Marine Chemical Factory, P. R. China), Sephadex LH-20 (GE Healthcare Bio-Xciences AB), and MCI gel CHP-20P (75–150 μm, Mitsubishi Chemical Corp., Tokyo, Japan). TLC was carried out on precoated silica gel GF254 glass plates. Spots were visualized under UV light and by dipping with 15 % H_2_SO_4_ in ethanol followed by heating. All solvents including petroleum ether (60–90 °C) were distilled prior to use.

### Plant Material

The fresh roots of *Euphorbia jolkinii* (or *Euphorbia nematocypha*) were collected from the alpine grasslands of Zhongdian county, Yunnan province, China, in June 2012, and were identified by Dr. Jian Liu. A voucher specimen (KIB20120620) has been deposited at the State Key Laboratory of Phytochemistry and Plant Resources in West China, Kunming Institute of Botany, Chinese Academy of Sciences.

### Extraction and Isolation

The fresh roots of *E. jolkinii* (27 kg) were chopped and extracted with MeOH (25 L × 3, each time 24 h) at room temperature. The combined extracts were evaporated to dryness under reduced pressure to obtain a crude extract (3470 g), which was suspended in H_2_O (4 L) and then extracted with petroleum ether (PE, 4 L × 4) and EtOAc (4 L × 4), successively, to yield a PE-soluble fraction (67.1 g) and an EtOAc-soluble fraction (61.6 g). The PE-soluble fraction (67.1 g) was subjected to silica gel column chromatography eluting with a gradient solvent system of chloroform/acetone (from 10:0 to 0:10, v/v) to afford six fractions (A–F) on the basis of TLC detection. Fraction A (30.8 g, CHCl_3_/Me_2_CO, 10:0) was chromatographed on silica gel columns, and then purified using a Sephadex LH-20 column (CHCl_3_/MeOH, 1:1, v/v) to yield compounds **13** (10 mg), **15** (25 mg), and **17** (6 mg). Fraction C (6.6 g, CHCl_3_/Me_2_CO, 8:2) was further chromatographed over a silica gel column using PE/Me_2_CO (9:2, v/v) as eluents to give four subfractions C1–C4. Subfraction C2 (560 mg) was purified by silica gel (PE/2-propanol, 30:1, v/v) and Sephadex LH-20 (CHCl_3_/MeOH, 1:1, v/v) columns to afford **3** (34 mg) and **10** (40 mg). Subfraction C3 (3.7 g) was repeatedly chromatographed on a Sephadex-LH_20_ column (Me_2_CO) and purified by reversed-phase semipreparative HPLC using 69 % MeOH in water as eluents (flow rate: 3 mL/min; column: ZORBAX SB-C_18_, 5 μm, 9.4 × 250 mm; detection: UV 280 nm; retention times: 12.1 and 15.3 min respectively) to obtain **1** (16 mg) and **2** (12 mg). Similarly, the EtOAc-soluble fraction (61.6 g) was separated by column chromatography over silica gel with a mixture of chloroform and acetone (from 10:0 to 0:10, v/v) to give six fractions a-f. Fraction b (5.0 g, CHCl_3_/Me_2_CO, 9:1) was chromatographed on silica gel and Sephadex-LH_20_ columns (CHCl_3_/MeOH, 1:1, v/v) to yield **6** (6 mg), **9** (75 mg), **19** (33 mg), **20** (4 mg), and **21** (2 mg). Fraction c (1.4 g) was subjected to silica gel column eluted with PE/Me_2_CO (40:1, v/v) to afford **8** (3 mg) and **18** (2 mg). The remaining subfractions were enriched (19.6 g) and were passed through a MCI gel column (MeOH/H_2_O, from 6:4 to 10:0) to give six subfractions (a1–a6). Subfraction a2 (2.3 g) was further separated with silica gel columns with PE and isopropyl alcohol (20:1, v/v) as eluents to afford **5** (4 mg), **7** (6 mg), **11** (6 mg), **12** (8 mg), **14** (4 mg), **16** (7 mg), and 37 mg of crude sample mainly containing **4**. Compound **4** (6.3 mg) was achieved by further purification with reversed-phase semipreparative HPLC using 80 % MeOH in water as eluents.

### 20-*O*-[(2′*E*,4′*E*)-6-oxo-2′,4′-Hexadienoyl] ingenol (**1**)

White soild; [*α*]_D_^17^ + 11.1 (*c* 0.1, MeOH); UV (MeOH) *λ*_max_ (log *ε*) 267 (3.15), and 202 (3.05) nm; IR (KBr) ν_max_ 3434, 2954, 2940, 2871, 1719, 1686, 1637, 1450, 1382, 1226, 1177, 1103, 1015 cm^−1^; ^1^H and ^13^C NMR: see Table 1; EIMS *m/z* 456 [M]^+^ (6), 411 (13), 368 (13), 330 (77), 135 (43), 121 (89), 91 (100), 77 (100), 67 (88), 55 (99); HREIMS *m/z* 456.2169 (calcd for C_26_H_32_O_7_, 456.2148).

### 20-*O*-[(2′*E*,4′*E*,6′*E*)-8-*oxo*-2′,4′,6′-octatrienoyl] ingenol (**2**)

White soild; [*α*]_D_^17^ + 18.2 (*c* 0.1, MeOH); UV (MeOH) *λ*_max_ (log *ε*) 280 (3.03), and 203 (3.16) nm; IR (KBr) ν_max_ 3433, 2955, 2925, 2872, 1716, 1638, 1381, 1149, 1016 cm^−1^; ^1^H and ^13^C NMR: see Table 1; EIMS *m/z* 482 [M]^+^ (10), 464 (12), 312 (15), 241 (18), 135 (55), 121 (64), 91 (72), 77 (100); HREIMS *m/z* 482.2301 (calcd for C_28_H_34_O_7_, 482.2305).

### (3*β*,12*β*)-3,12-Dihydroxyisopimara-7,15-dien-2-one (**4**)

Colorless needles; [*α*]_D_^17^ + 29.4 (*c* 0.3, MeOH); UV (MeOH) *λ*_max_ (log *ε*) 203 (2.75) nm; IR (KBr) ν_max_ 3442, 2968, 2924, 1716, 1632, 1391, 1112, 1059 cm^−1^; ^1^H and ^13^C NMR: see Table 1; EIMS *m/z* 318 [M]^+^ (38), 300 (100), 285 (23), 227 (35), 185 (43), 164 (44), 105 (54), 55 (50); HREIMS *m/z* 318.2194 (calcd for C_20_H_30_O_3_, 318.2195).

### (3*β*,12*β*)-3,12-Dihydroxyisopimara-7,15-diene (**5**)

Colorless needles; [*α*]_D_^17^ – 26.0 (*c* 0.2, MeOH); UV (MeOH) *λ*_max_ (log *ε*) 203 (2.74) nm; IR (KBr) ν_max_ 3441, 3425, 2962, 2928, 2853, 1635, 1384, 1088, 1039, 997, 915 cm^−1^; ^1^H and ^13^C NMR: see Table 2; EIMS *m/z* 304 [M]^+^ (8), 286 (100), 268 (43), 253 (65), 146 (51), 105 (41), 55 (47); HREIMS *m/z* 304.2401 (calcd for C_20_H_32_O_2_, 304.2402).

### (12*β*)-2,12-Dihydroxyisopimara-1,7,15-trien-3-one (**6**)

Colorless oil; [*α*]_D_^17^ + 9.0 (*c* 0.2, MeOH); UV (MeOH) *λ*_max_ (log *ε*) 266 (2.64), and 203 (2.84) nm; IR (KBr) ν_max_ 3433, 2967, 2925, 2873, 1714, 1673, 1651, 1408, 1221, 1052 cm^−1^; ^1^H and ^13^C NMR: see Table 2; EIMS *m/z* 316 [M]^+^ (6), 288 (7), 152 (100), 124 (98), 109 (51), 83 (73), 55 (58); HREIMS *m/z* 316.2038 (calcd for C_20_H_28_O_3_, 316.2038).

### *Ent*-3-oxoatis-16*α*,17-acetonide (**8**)

Colorless needles; [*α*]_D_^17^ – 44.7 (*c* 0.2, MeOH); UV (MeOH) *λ*_max_ (log *ε*) 228 (2.51), and 200 (2.42) nm; IR (KBr) ν_max_ 2982, 2935, 2869, 1706, 1674, 1451, 1381, 1370, 1261, 1212, 1053 cm^−1^; ^1^H and ^13^C NMR: see Table 2; positive ESIMS *m/z* 383 [M + Na]^+^; HREIMS *m/z* 360.2682 (calcd for C_23_H_36_O_3_, 360.2664).

### Antifeeding Assay

Beet armyworms (*Spodoptera exigua*) were purchased from the Pilot-Scale Base of Bio-Pesticides, Institute of Zoology, Chinese Academy of Sciences. A modified dual-choice bioassay as previously described was performed for antifeedant test [22, 23] The larvae were reared on an artificial diet in our laboratory under controlled photoperiod (light: dark, 12:8 h) and temperature (25 ± 2 °C). Larvae were starved 3–4 h prior to each bioassay. Fresh leaf discs were cut from *Brassica chinensis*, using a cork borer (1.1 cm in diameter). Treated leaf discs were painted with 10 μL of acetone solution containing the test compound, and control leaf discs with the same amount of acetone. After air drying, two tested leaf discs and two control ones were set in alternating position in the same Petri dish (90 mm in diameter), with moistened filter paper at the bottom. Two thirds of instars were placed at the center of the Petri dish. Five replicates were run for each treatment. After feeding for 24 h, areas of leaf discs consumed were measured. The insect antifeedant potency of the test compound was evaluated in terms of the EC_50_ value which was determined by Probit analysis for each insect species.

### Cytotoxicity Assay

Cytotoxicity assay was kindly carried out according to the established protocols [24, 25]. The Hep-2 cells (human larynx epidermoid carcinoma cell line) and Vero cells (African green monkey kidney cell line) were grown in 96-well plates (8 mm diameter; Falcon Plastics, Oxnard, CA) and incubated at 37 °C in a humidified atmosphere supplied with 5 % carbon dioxide. When the cell cultures were confluent, the culture medium was removed from each well and replenished with 0.1 mL of the maintenance medium. To test for cytotoxicity, 0.1 mL of the maintenance medium containing the tested samples in serial twofold dilutions was added to each of the wells. The well with 0.1 mL maintenance medium but without sample was prepared to act as a cell control. All the cultures were then incubated at 37 °C for 3 days. The morphology of the cells was inspected daily and observed microscopically for any detectable alterations. The 50 % cytotoxicity concentration (CC_50_), the concentration required to cause visible changes in 50 % of the intact cells, was evaluated. The maximal non-cytotoxic concentration (MNCC) was estimated as the maximal concentration of the samples that did not exert toxic effects by microscopic monitoring.

### Antivirus Assay

RSV (respiratory syncytial virus, long strain), Cox B3 (Coxsackie B3), and HSV-1 (Herpes simplex virus type 1, 15577 strain) were used for antivirus assay as methods described in the literature [24, 25]. A cytopathic effect (CPE) reduction assay was adopted for screening the antiviral activities of the pure compounds isolated from the roots of *E. jolkinii* in the present study. In brief, to confluent cell monolayers in a 96-well plate, 100 TCID_50_ (tissue culture medium infective dose) virus suspension and serial two-fold dilutions of compounds were added to each test well simultaneously. The virus suspension and dilution medium without samples were added, respectively, to the cell cultures to serve as the virus control and cell control. The plates were then incubated at 37 °C in a humidified CO_2_ atmosphere for 3–6 days. The RSV induced CPE was scored under light microscopy (score 0, 0 %; score 1, 0–25 %; score 2, 35–50 %; score 3, 50–75 %; and score 4, 75–100 %). The concentration that reduced 50 % of CPE in respect to the virus control was estimated from the plots of the data and was defined as the 50 % inhibitory concentration (IC_50_). The selective index (SI) was calculated from the ratio CC_50_/IC_50_ [26].

## Electronic Supplementary Material

Below is the link to the electronic supplementary material.

Supplementary material 1 (DOCX 1917 kb)
