# Supplementary material for: Antifeedant and Antiviral Diterpenoids from the Fresh Roots of Euphorbia jolkinii
Source: Nat Prod Bioprospect. 2014 Apr 9;4(2):91–100. doi: 10.1007/s13659-014-0009-3 (PMC4004854; doi:10.1007/s13659-014-0009-3)
Supplement: Supplementary file 1 — Supplementary material 1 (DOCX 1917 kb) [file 13659_2014_9_MOESM1_ESM.docx]

**Electronic Supplementary Material**

**Antifeedant and antiviral diterpenoids from fresh roots of *Euphorbia nematocypha***

Chun-Shuai HUANG,^a,c^ Shi-Hong LUO,^a^ Yao-Lan LI,^b^ Chun-Huan LI,^a^ Juan HUA,^a^ Yan LIU,^a^ Shu-Xi JING,^a^ Ying WANG,^a^ Min-Jie YANG,^a^ and Sheng-Hong LI ^a,*^

^a^State Key Laboratory of Phytochemistry and Plant Resources in West China, Kunming Institute of Botany, Chinese Academy of Sciences, Kunming 650201, China

^b^Institute of Traditional Chinese Medicine and Natural Products, Jinan University, Guangzhou 510632, China

^c^Graduate University of Chinese Academy of Sciences, Beijing 100049, China

^*^ To whom correspondence should be addressed. Tel/Fax: (+86) 871-65223035.

E-mail: [shli@mail.kib.ac.cn](mailto:shli@mail.kib.ac.cn)

| **Table of Contents** | |  |
| --- | --- | --- |
| ***Table S1.*** Key ROESY correlations of **4**-**6**. | P. 3 | |
| ***Fig. S1.*** ^1^H NMR spectrum of **1** recorded at 400 MHz in acetone-*d*_6_. | P. 4 | |
| ***Fig. S2.*** ^13^C NMR and DEPT spectra of **1** recorded at 100 MHz in acetone-*d*_6_. | P. 4 | |
| ***Fig. S3.*** ^1^H NMR spectrum of **2** recorded at 400 MHz in acetone-*d*_6_. | P. 5 | |
| ***Fig. S4.*** ^13^C NMR and DEPT spectra of **2** recorded at 100 MHz in acetone-*d*_6_. | P. 5 | |
| ***Fig. S5.*** ^1^H NMR spectrum of **4** recorded at 400 MHz in acetone-*d*_6_. | P. 6 | |
| ***Fig. S6.*** ^13^C NMR and DEPT spectra of **4** recorded at 100 MHz in acetone-*d*_6_. | P. 6 | |
| ***Fig. S7.*** ^1^H NMR spectrum of **5** recorded at 600 MHz in acetone-*d*_6_. | P. 7 | |
| ***Fig. S8.*** ^13^C NMR and DEPT spectra of **5** recorded at 150 MHz in acetone-*d*_6_. | P. 7 | |
| ***Fig. S9.*** ^1^H NMR spectrum of **6** recorded at 400 MHz in CDCl_3_. | P. 8 | |
| ***Fig. S10.*** ^13^C NMR and DEPT spectra of **6** recorded at 100 MHz in CDCl_3_. | P. 8 | |
| ***Fig. S11.*** ^1^H NMR spectrum of **8** recorded at 400 MHz in acetone-*d*_6_. | P. 9 | |
| ***Fig. S12.*** ^13^C NMR and DEPT spectra of **8** recorded at 100 MHz in acetone-*d*_6_. | P. 9 | |

**Table S1.** Key ROESY correlations of **4**-**6**.

| No. | **4** | **5** | **6** |
| --- | --- | --- | --- |
| H-3 | H-5, Me-18 | H-1*α*, H-5, Me-18 |  |
| H-5 | H-3, H-9, H-11*α*, Me-18 | H-3, H-9 | H-9, Me-18 |
| H-6*α* |  |  | Me-18 |
| H-6*β* |  |  | Me-19 |
| H-9 | H-5, H-11*α*, H-12 | H-5, H-14*α* | H-5, H-12 |
| H-11*α* | H-9, H-11*β*, H-12 |  | H-9, H-12 |
| H-11*β* | H-11*α*, Me-17, Me-20 |  | Me-17, Me-20 |
| H-12 | H-9, H-11*α* | H-15 | H-9, H-11*α*, H-15 |
| H-15 | H-12, H-16 | H-14*α*, H_2_-16 | H-12, H_2_-14, H_2_-16 |
| Me-17 | H-11*β* |  | H-11*β*, H_2_-14, Me-19 |
| Me-18 | H-5 | H-3, H-5 | H-5 |
| Me-19 | Me-20 |  | H-6*β* |
| Me-20 | H_2_-1, H-11*β*, Me-19 | H-1*β* | H-6*β*, H-11*β* |
| 3-OH | Me-19 | Me-19 |  |
| 12-OH |  | H-14*β* |  |





| ***Fig. S1.*** ^1^H NMR spectrum of **1** recorded at 400 MHz in acetone-*d*_6_. |
| --- |





***Fig. S2.*** ^13^C NMR and DEPT spectra of **1** recorded at 100 MHz in acetone-*d*_6_.





| ***Fig. S3.*** ^1^H NMR spectrum of **2** recorded at 400 MHz in acetone-*d*_6_. |
| --- |





***Fig. S4.*** ^13^C NMR and DEPT spectra of **2** recorded at 100 MHz in acetone-*d*_6_.





***Fig. S5.*** ^1^H NMR spectrum of **4** recorded at 400 MHz in acetone-*d*_6_.



***Fig. S6.*** ^13^C NMR and DEPT spectra of **4** recorded at 100 MHz in acetone-*d*_6_.





| ***Fig. S7.*** ^1^H NMR spectrum of **5** recorded at 600 MHz in acetone-*d*_6_. |
| --- |





***Fig. S8.*** ^13^C NMR and DEPT spectra of **5** recorded at 150 MHz in acetone-*d*_6_.





| ***Fig. S9.*** ^1^H NMR spectrum of **6** recorded at 400 MHz in CDCl_3_. |
| --- |





***Fig. S10.*** ^13^C NMR and DEPT spectra of **6** recorded at 100 MHz in CDCl_3_.





| ***Fig. S11.*** ^1^H NMR spectrum of **8** recorded at 400 MHz in acetone-*d*_6_. |
| --- |





***Fig. S12.*** ^13^C NMR and DEPT spectra of **8** recorded at 100 MHz in acetone-*d*_6_
